# 4-(4-Bromo­benzyl­ideneamino)-1-(diphenyl­amino­meth­yl)-3-[1-(4-isobutyl­phen­yl)eth­yl]-1*H*-1,2,4-triazole-5(4*H*)-thione

**DOI:** 10.1107/S1600536808012713

**Published:** 2008-05-07

**Authors:** Hoong-Kun Fun, Samuel Robinson Jebas, K. V. Sujith, P. S. Patil, B. Kalluraya, S. M Dharmaprakash

**Affiliations:** a X-ray Crystallography Unit, School of Physics, Universiti Sains Malaysia, 11800 USM, Penang, Malaysia; b Department of Studies in Chemistry, Mangalore University, Mangalagangotri, Mangalore 574 199, India; c Department of Studies in Physics, Mangalore University, Mangalagangotri, Mangalore 574 199, India

## Abstract

In the title compound, C_34_H_34_BrN_5_S, the two phenyl rings of the diphenyl­amino­methyl group are inclined at an angle of 73.86 (8)° and they form dihedral angles of 74.04 (8) and 48.74 (8)° with the triazole ring. Intra­molecular C—H⋯S hydrogen bonds generate *S*(6) and *S*(5) ring motifs. The crystal structure is stabilized by weak C—H⋯π inter­actions.

## Related literature

For related literature, see: Dave *et al.* (2007 ▶); Kalluraya *et al.* (2003 ▶, 2004 ▶, 2007 ▶); Kane *et al.* (1990 ▶). For literature on Mannich bases, see: Kalluraya *et al.* (2001 ▶). For bond-length data, see: Allen *et al.* (1987 ▶). For related literature on hydrogen-bond motifs, see: Bernstein *et al.* (1995 ▶).
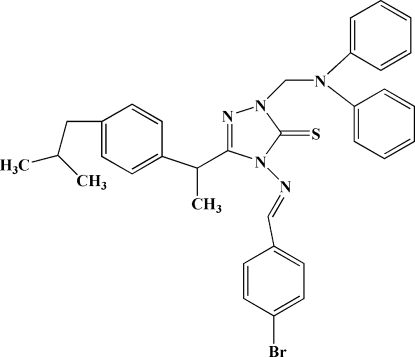


## Experimental

### 

#### Crystal data

C_34_H_34_BrN_5_S
*M* *_r_* = 624.63
Monoclinic, 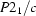

*a* = 10.9672 (1) Å
*b* = 9.7833 (1) Å
*c* = 28.6210 (3) Å
β = 105.966 (1)°
*V* = 2952.44 (5) Å^3^
*Z* = 4
Mo *K*α radiation
μ = 1.50 mm^−1^
*T* = 100.0 (1) K
0.35 × 0.31 × 0.27 mm

#### Data collection

Bruker SMART APEXII CCD area-detector diffractometer
Absorption correction: multi-scan (*SADABS*; Bruker, 2005 ▶) *T*_min_ = 0.623, *T*_max_ = 0.684
55283 measured reflections
13248 independent reflections
7914 reflections with *I* > 2σ(*I*)
*R*_int_ = 0.059

#### Refinement

*R*[*F* ^2^ > 2σ(*F* ^2^)] = 0.044
*wR*(*F* ^2^) = 0.098
*S* = 1.01
13248 reflections
372 parameters
H-atom parameters constrained
Δρ_max_ = 0.46 e Å^−3^
Δρ_min_ = −0.57 e Å^−3^

### 

Data collection: *APEX2* (Bruker, 2005 ▶); cell refinement: *APEX2*; data reduction: *SAINT* (Bruker, 2005 ▶); program(s) used to solve structure: *SHELXTL* (Sheldrick, 2008 ▶); program(s) used to refine structure: *SHELXTL* molecular graphics: *SHELXTL*; software used to prepare material for publication: *SHELXTL* and *PLATON* (Spek, 2003 ▶).

## Supplementary Material

Crystal structure: contains datablocks global, I. DOI: 10.1107/S1600536808012713/ci2592sup1.cif

Structure factors: contains datablocks I. DOI: 10.1107/S1600536808012713/ci2592Isup2.hkl

Additional supplementary materials:  crystallographic information; 3D view; checkCIF report

## Figures and Tables

**Table 1:** Hydrogen-bond geometry (Å, °)

*D*—H⋯*A*	*D*—H	H⋯*A*	*D*⋯*A*	*D*—H⋯*A*
C3—H3*A*⋯S1	0.93	2.52	3.217 (2)	132
C22—H22*B*⋯S1	0.97	2.80	3.232 (2)	108
C6—H6*A*⋯*Cg*1^i^	0.93	2.81	3.717 (2)	165
C21—H21*C*⋯*Cg*2^ii^	0.96	2.89	3.829 (2)	168

Symmetry codes: (i) 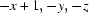; (ii) 
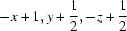
. *Cg*1 and *Cg*2 are the centroids of the C11–C16 and C23–C28 rings, respectively.

**Fig. 1.**
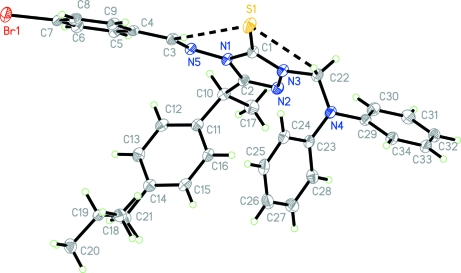
The molecular structure of the title compound, showing 50% probability displacement ellipsoids and the atom-numbering scheme. Dashed lines indicate hydrogen bonds.

## supplementary crystallographic information

### Comment

Nitrogen-containing heterocyclic molecules constitute the largest portion of chemical entities, which are part of many natural products, fine chemicals, and biologically active pharmaceuticals vital for enhancing the quality of life (Kalluraya *et al.* 2003, 2007; Kane *et al.*, 1990). Mannich bases are a class of heterocycles, which have attracted significant interest in medicinal chemistry (Kalluraya *et al.*, 2004). Among the Mannich bases, 1,2,4-triazole derivatives have attracted considerable attention because of their wide variety of biological activities, such as antineoplastic, analgesic and antibiotic activity (Dave *et al.*, 2007). Mannich bases are obtained by condensing an amine, formaldehyde and a compound containing active hydrogen atom (Kalluraya *et al.*, 2001). It is interesting to note that the reaction is highly regioselective and furnishes only the N-Mannich base and none of the S-Mannich derivatives, though the intermediate Schiff bases can exist in the thiol-thione tautomeric equilibrium. In view of these impressive array of properties exhibited by Mannich bases, the crystal structure of the title compound is reported here.

Bond lengths and angles in the title compound have normal values (Allen *et al.*, 1987). The triazole ring is planar with a maximium deviation of 0.010 (2) Å for atom C1. The planes through the C4—C9, C11—C16, C23—C28 and C29—C34 rings form dihedral angles of 25.10 (8), 81.35 (8), 74.04 (8) and 48.74 (8)°, respectively, with the the triazole ring. Weak C—H···S hydrogen bonds generating S(6) and S(5) ring motifs (Bernstein *et al.*,(1995) are observed in the molecular structure.

The crystal packing is stabilized by weak C—H···π interactions involving the C11–C16 (centroid *Cg*1) and C23–C28 (centroid *Cg*2) rings (Table 1).

### Experimental

The title compound, a Mannich base, was obtained by the aminomethylation of a Schiff base, 4-{[(4-bromophenyl)methylene]amino}-5-[1-(4-isobutylphenyl)ethyl]-3-mercapto- 1,2,4-triazole which was in turn obtained by refluxing 4-amino-3-mercapto-5- [1-(4-isobutylphenyl)ethyl]-1,2,4-triazole (0.01 mol) and 4-bromo benzaldehyde (0.01 mol) in ethanol (30 ml) by adding 2 drops of concentrated sulfuric acid for 3 h. A mixture of the obtained Schiff base (0.01 mol), formaldehyde (40%, 1 ml) and diphenyl amine (0.01 mol) in ethanol (50 ml) was stirred at room temperature for 16 h. The solid product was collected by filtration, washed with ethanol and dried. It was then recrystallized from ethanol. Crystals suitable for X-ray analysis were obtained from an acetone-*N*,*N*-dimethylformamide (DMF) (1:3) solution by slow evaporation (yield 68%; m.p. 381–382 K). Analysis (%) for C_34_H_34_N_5_BrS found (calculated): C 65.23 (65.38), H 5.33 (5.44), N 11.21 (11.17).

### Refinement

H atoms were positioned geometrically [C–H = 0.93–0.98 Å] and refined using a riding model, with *U*_iso_(H) = 1.2–1.5*U*_eq_(C). A rotating-group model was used for the methyl groups.

### Crystal data

**Table:**

C_34_H_34_BrN_5_S	*F*_000_ = 1296
*M**_r_* = 624.63	*D*_x_ = 1.405 Mg m^−^^3^
Monoclinic, *P*2_1_/*c*	Mo *K*α radiation λ = 0.71073 Å
Hall symbol: -P 2ybc	Cell parameters from 8665 reflections
*a* = 10.9672 (1) Å	θ = 2.2–29.2º
*b* = 9.7833 (1) Å	µ = 1.50 mm^−^^1^
*c* = 28.6210 (3) Å	*T* = 100.0 (1) K
β = 105.966 (1)º	Block, colourless
*V* = 2952.44 (5) Å^3^	0.35 × 0.31 × 0.27 mm
*Z* = 4	

### Data collection

**Table:**

Bruker SMART APEXII CCD area-detector diffractometer	13248 independent reflections
Radiation source: fine-focus sealed tube	7914 reflections with *I* > 2σ(*I*)
Monochromator: graphite	*R*_int_ = 0.059
*T* = 100.0(1) K	θ_max_ = 35.4º
φ and ω scans	θ_min_ = 1.9º
Absorption correction: multi-scan(SADABS; Bruker, 2005)	*h* = −17→17
*T*_min_ = 0.623, *T*_max_ = 0.684	*k* = −15→15
55283 measured reflections	*l* = −46→46

### Refinement

**Table:**

Refinement on *F*^2^	Secondary atom site location: difference Fourier map
Least-squares matrix: full	Hydrogen site location: inferred from neighbouring sites
*R*[*F*^2^ > 2σ(*F*^2^)] = 0.044	H-atom parameters constrained
*wR*(*F*^2^) = 0.098	*w* = 1/[σ^2^(*F*_o_^2^) + (0.0352*P*)^2^ + 0.5449*P*] where *P* = (*F*_o_^2^ + 2*F*_c_^2^)/3
*S* = 1.02	(Δ/σ)_max_ = 0.001
13248 reflections	Δρ_max_ = 0.46 e Å^−^^3^
372 parameters	Δρ_min_ = −0.57 e Å^−^^3^
Primary atom site location: structure-invariant direct methods	Extinction correction: none

### Special details

**Table:**

Experimental. The data was collected with the Oxford Cyrosystem Cobra low-temperature attachment.
Geometry. All e.s.d.'s (except the e.s.d. in the dihedral angle between two l.s. planes) are estimated using the full covariance matrix. The cell e.s.d.'s are taken into account individually in the estimation of e.s.d.'s in distances, angles and torsion angles; correlations between e.s.d.'s in cell parameters are only used when they are defined by crystal symmetry. An approximate (isotropic) treatment of cell e.s.d.'s is used for estimating e.s.d.'s involving l.s. planes.
Refinement. Refinement of *F*^2^ against ALL reflections. The weighted *R*-factor *wR* and goodness of fit *S* are based on *F*^2^, conventional *R*-factors *R* are based on *F*, with *F* set to zero for negative *F*^2^. The threshold expression of *F*^2^ > σ(*F*^2^) is used only for calculating *R*-factors(gt) *etc*. and is not relevant to the choice of reflections for refinement. *R*-factors based on *F*^2^ are statistically about twice as large as those based on *F*, and *R*- factors based on ALL data will be even larger.

### Fractional atomic coordinates and isotropic or equivalent isotropic displacement parameters (Å^2^)

**Table:**

	*x*	*y*	*z*	*U*_iso_*/*U*_eq_	
Br1	0.543981 (15)	−0.004817 (17)	−0.133261 (6)	0.02529 (5)	
S1	0.15515 (5)	−0.27622 (4)	0.091481 (16)	0.02758 (10)	
N1	0.11707 (12)	−0.00549 (12)	0.06174 (4)	0.0177 (2)	
N2	0.01822 (12)	0.07970 (13)	0.11283 (5)	0.0194 (3)	
N3	0.05163 (13)	−0.05530 (13)	0.12340 (4)	0.0192 (3)	
N4	0.05997 (13)	−0.03122 (13)	0.20801 (5)	0.0213 (3)	
N5	0.16650 (12)	0.00579 (12)	0.02217 (4)	0.0186 (2)	
C1	0.11076 (15)	−0.11338 (15)	0.09263 (5)	0.0194 (3)	
C2	0.05841 (14)	0.10666 (15)	0.07525 (5)	0.0176 (3)	
C3	0.25133 (14)	−0.08055 (15)	0.01851 (5)	0.0191 (3)	
H3A	0.2737	−0.1521	0.0406	0.023*	
C4	0.31254 (14)	−0.06601 (15)	−0.02038 (5)	0.0170 (3)	
C5	0.41316 (15)	−0.15262 (15)	−0.02088 (5)	0.0193 (3)	
H5A	0.4342	−0.2231	0.0017	0.023*	
C6	0.48243 (15)	−0.13606 (15)	−0.05424 (5)	0.0194 (3)	
H6A	0.5502	−0.1935	−0.0539	0.023*	
C7	0.44857 (15)	−0.03203 (15)	−0.08809 (5)	0.0194 (3)	
C8	0.34706 (15)	0.05398 (16)	−0.08930 (5)	0.0202 (3)	
H8A	0.3250	0.1224	−0.1127	0.024*	
C9	0.27910 (15)	0.03763 (15)	−0.05571 (5)	0.0195 (3)	
H9A	0.2111	0.0951	−0.0564	0.023*	
C10	0.04978 (14)	0.24247 (15)	0.05043 (5)	0.0196 (3)	
H10A	0.0236	0.2270	0.0152	0.023*	
C11	0.17723 (14)	0.31489 (14)	0.06341 (5)	0.0181 (3)	
C12	0.21998 (15)	0.37842 (15)	0.02736 (5)	0.0203 (3)	
H12A	0.1743	0.3683	−0.0050	0.024*	
C13	0.32967 (16)	0.45656 (16)	0.03905 (6)	0.0220 (3)	
H13A	0.3565	0.4979	0.0143	0.026*	
C14	0.40058 (15)	0.47425 (15)	0.08733 (6)	0.0198 (3)	
C15	0.35851 (15)	0.40810 (16)	0.12319 (6)	0.0218 (3)	
H15A	0.4047	0.4170	0.1556	0.026*	
C16	0.24913 (15)	0.32926 (16)	0.11149 (5)	0.0208 (3)	
H16A	0.2235	0.2855	0.1361	0.025*	
C17	−0.05057 (16)	0.33197 (16)	0.06350 (6)	0.0258 (3)	
H17A	−0.1306	0.2851	0.0550	0.039*	
H17B	−0.0586	0.4165	0.0459	0.039*	
H17C	−0.0254	0.3504	0.0978	0.039*	
C18	0.51856 (15)	0.56133 (16)	0.09975 (6)	0.0228 (3)	
H18A	0.5288	0.6009	0.1317	0.027*	
H18B	0.5065	0.6360	0.0766	0.027*	
C19	0.64115 (15)	0.48568 (15)	0.09976 (5)	0.0193 (3)	
H19A	0.6274	0.4395	0.0683	0.023*	
C20	0.74986 (16)	0.58645 (16)	0.10501 (6)	0.0262 (3)	
H20A	0.7281	0.6517	0.0790	0.039*	
H20B	0.8250	0.5380	0.1038	0.039*	
H20C	0.7650	0.6332	0.1356	0.039*	
C21	0.67544 (15)	0.37717 (16)	0.13955 (6)	0.0227 (3)	
H21A	0.7493	0.3281	0.1371	0.034*	
H21B	0.6059	0.3147	0.1357	0.034*	
H21C	0.6927	0.4204	0.1708	0.034*	
C22	0.02950 (16)	−0.12061 (16)	0.16644 (5)	0.0224 (3)	
H22A	−0.0588	−0.1475	0.1593	0.027*	
H22B	0.0808	−0.2026	0.1740	0.027*	
C23	0.18209 (15)	0.02413 (15)	0.22580 (5)	0.0196 (3)	
C24	0.29008 (16)	−0.04544 (17)	0.22172 (6)	0.0227 (3)	
H24A	0.2821	−0.1297	0.2061	0.027*	
C25	0.40981 (16)	0.01206 (17)	0.24111 (6)	0.0258 (3)	
H25A	0.4816	−0.0355	0.2391	0.031*	
C26	0.42321 (16)	0.13885 (18)	0.26334 (6)	0.0275 (4)	
H26A	0.5032	0.1768	0.2761	0.033*	
C27	0.31547 (17)	0.20828 (17)	0.26626 (6)	0.0275 (4)	
H27A	0.3236	0.2940	0.2808	0.033*	
C28	0.19685 (15)	0.15264 (16)	0.24802 (6)	0.0231 (3)	
H28A	0.1258	0.2009	0.2505	0.028*	
C29	−0.03703 (14)	0.00563 (15)	0.22995 (5)	0.0183 (3)	
C30	−0.14889 (15)	0.06584 (17)	0.20375 (6)	0.0234 (3)	
H30A	−0.1623	0.0840	0.1708	0.028*	
C31	−0.24132 (15)	0.09931 (17)	0.22671 (6)	0.0252 (3)	
H31A	−0.3170	0.1389	0.2090	0.030*	
C32	−0.22099 (15)	0.07391 (16)	0.27591 (6)	0.0238 (3)	
H32A	−0.2830	0.0962	0.2912	0.029*	
C33	−0.10801 (15)	0.01518 (15)	0.30232 (6)	0.0222 (3)	
H33A	−0.0938	−0.0006	0.3354	0.027*	
C34	−0.01663 (15)	−0.01996 (15)	0.27969 (6)	0.0207 (3)	
H34A	0.0585	−0.0606	0.2974	0.025*	

### Atomic displacement parameters (Å^2^)

**Table:**

	*U*^11^	*U*^22^	*U*^33^	*U*^12^	*U*^13^	*U*^23^
Br1	0.02348 (8)	0.03478 (9)	0.01983 (7)	0.00250 (7)	0.00969 (6)	0.00190 (7)
S1	0.0432 (3)	0.01778 (18)	0.0276 (2)	0.00609 (17)	0.01949 (19)	0.00226 (15)
N1	0.0210 (6)	0.0184 (6)	0.0148 (5)	0.0036 (5)	0.0069 (5)	0.0005 (5)
N2	0.0205 (6)	0.0183 (6)	0.0200 (6)	0.0040 (5)	0.0064 (5)	0.0002 (5)
N3	0.0237 (6)	0.0183 (6)	0.0171 (6)	0.0025 (5)	0.0079 (5)	0.0003 (5)
N4	0.0200 (6)	0.0272 (7)	0.0181 (6)	−0.0008 (5)	0.0076 (5)	−0.0047 (5)
N5	0.0207 (6)	0.0199 (6)	0.0161 (5)	−0.0001 (5)	0.0066 (5)	−0.0017 (5)
C1	0.0221 (7)	0.0199 (7)	0.0168 (7)	0.0015 (6)	0.0067 (6)	−0.0008 (5)
C2	0.0165 (7)	0.0195 (7)	0.0168 (6)	0.0023 (5)	0.0044 (6)	−0.0018 (5)
C3	0.0225 (7)	0.0192 (7)	0.0156 (6)	0.0008 (6)	0.0052 (6)	0.0002 (5)
C4	0.0188 (7)	0.0173 (7)	0.0144 (6)	−0.0004 (5)	0.0037 (5)	−0.0014 (5)
C5	0.0251 (8)	0.0165 (7)	0.0160 (6)	0.0018 (6)	0.0053 (6)	−0.0006 (5)
C6	0.0208 (7)	0.0178 (7)	0.0193 (7)	0.0025 (6)	0.0051 (6)	−0.0032 (5)
C7	0.0209 (7)	0.0231 (7)	0.0146 (6)	−0.0007 (6)	0.0056 (6)	−0.0030 (5)
C8	0.0215 (7)	0.0212 (7)	0.0170 (7)	0.0027 (6)	0.0037 (6)	0.0018 (6)
C9	0.0197 (7)	0.0206 (7)	0.0175 (7)	0.0033 (6)	0.0040 (6)	−0.0008 (5)
C10	0.0209 (7)	0.0191 (7)	0.0178 (7)	0.0040 (6)	0.0036 (6)	0.0001 (5)
C11	0.0206 (7)	0.0151 (6)	0.0191 (7)	0.0051 (5)	0.0062 (6)	0.0004 (5)
C12	0.0242 (7)	0.0200 (7)	0.0151 (6)	0.0052 (6)	0.0028 (6)	0.0019 (5)
C13	0.0256 (8)	0.0210 (7)	0.0200 (7)	0.0043 (6)	0.0075 (6)	0.0048 (6)
C14	0.0222 (7)	0.0162 (7)	0.0212 (7)	0.0043 (5)	0.0061 (6)	0.0011 (5)
C15	0.0239 (8)	0.0240 (8)	0.0172 (7)	0.0031 (6)	0.0052 (6)	−0.0019 (6)
C16	0.0245 (8)	0.0220 (7)	0.0175 (7)	0.0024 (6)	0.0088 (6)	0.0013 (6)
C17	0.0220 (8)	0.0230 (8)	0.0317 (9)	0.0060 (6)	0.0059 (7)	0.0004 (7)
C18	0.0265 (8)	0.0180 (7)	0.0239 (8)	0.0008 (6)	0.0068 (6)	0.0011 (6)
C19	0.0242 (7)	0.0185 (7)	0.0163 (6)	−0.0004 (6)	0.0074 (6)	−0.0008 (5)
C20	0.0297 (9)	0.0208 (8)	0.0320 (9)	0.0001 (7)	0.0151 (7)	0.0009 (7)
C21	0.0251 (8)	0.0213 (7)	0.0213 (7)	0.0005 (6)	0.0059 (6)	0.0012 (6)
C22	0.0266 (8)	0.0234 (8)	0.0191 (7)	−0.0006 (6)	0.0099 (6)	−0.0016 (6)
C23	0.0218 (7)	0.0220 (8)	0.0164 (6)	0.0015 (6)	0.0074 (6)	0.0027 (5)
C24	0.0265 (8)	0.0241 (7)	0.0201 (7)	0.0031 (6)	0.0108 (6)	0.0007 (6)
C25	0.0218 (7)	0.0350 (9)	0.0231 (7)	0.0051 (7)	0.0106 (6)	0.0053 (7)
C26	0.0216 (8)	0.0352 (9)	0.0270 (8)	−0.0035 (7)	0.0088 (7)	0.0029 (7)
C27	0.0297 (9)	0.0237 (8)	0.0301 (9)	−0.0030 (7)	0.0101 (7)	0.0006 (7)
C28	0.0218 (7)	0.0215 (8)	0.0278 (8)	0.0019 (6)	0.0102 (7)	0.0033 (6)
C29	0.0196 (6)	0.0181 (7)	0.0179 (6)	0.0001 (6)	0.0062 (5)	−0.0011 (6)
C30	0.0260 (8)	0.0258 (8)	0.0171 (7)	0.0016 (7)	0.0038 (6)	0.0019 (6)
C31	0.0194 (7)	0.0266 (8)	0.0280 (8)	0.0029 (6)	0.0035 (6)	0.0025 (7)
C32	0.0200 (7)	0.0243 (8)	0.0299 (8)	0.0005 (6)	0.0116 (6)	−0.0030 (7)
C33	0.0254 (8)	0.0246 (8)	0.0182 (7)	−0.0008 (6)	0.0087 (6)	0.0004 (6)
C34	0.0203 (7)	0.0231 (8)	0.0180 (7)	0.0030 (6)	0.0042 (6)	0.0020 (6)

### Geometric parameters (Å, °)

**Table:**

Br1—C7	1.8925 (16)	C17—H17A	0.96
S1—C1	1.6688 (15)	C17—H17B	0.96
N1—C2	1.3800 (19)	C17—H17C	0.96
N1—N5	1.3879 (18)	C18—C19	1.535 (2)
N1—C1	1.3907 (19)	C18—H18A	0.97
N2—C2	1.2968 (19)	C18—H18B	0.97
N2—N3	1.3818 (18)	C19—C20	1.522 (2)
N3—C1	1.354 (2)	C19—C21	1.526 (2)
N3—C22	1.466 (2)	C19—H19A	0.98
N4—C23	1.404 (2)	C20—H20A	0.96
N4—C29	1.422 (2)	C20—H20B	0.96
N4—C22	1.4399 (19)	C20—H20C	0.96
N5—C3	1.2821 (19)	C21—H21A	0.96
C2—C10	1.497 (2)	C21—H21B	0.96
C3—C4	1.456 (2)	C21—H21C	0.96
C3—H3A	0.93	C22—H22A	0.97
C4—C5	1.395 (2)	C22—H22B	0.97
C4—C9	1.408 (2)	C23—C28	1.398 (2)
C5—C6	1.383 (2)	C23—C24	1.398 (2)
C5—H5A	0.93	C24—C25	1.396 (2)
C6—C7	1.384 (2)	C24—H24A	0.93
C6—H6A	0.93	C25—C26	1.383 (2)
C7—C8	1.388 (2)	C25—H25A	0.93
C8—C9	1.378 (2)	C26—C27	1.385 (2)
C8—H8A	0.93	C26—H26A	0.93
C9—H9A	0.93	C27—C28	1.374 (2)
C10—C11	1.519 (2)	C27—H27A	0.93
C10—C17	1.531 (2)	C28—H28A	0.93
C10—H10A	0.98	C29—C30	1.381 (2)
C11—C12	1.392 (2)	C29—C34	1.402 (2)
C11—C16	1.392 (2)	C30—C31	1.390 (2)
C12—C13	1.386 (2)	C30—H30A	0.93
C12—H12A	0.93	C31—C32	1.386 (2)
C13—C14	1.397 (2)	C31—H31A	0.93
C13—H13A	0.93	C32—C33	1.386 (2)
C14—C15	1.395 (2)	C32—H32A	0.93
C14—C18	1.508 (2)	C33—C34	1.378 (2)
C15—C16	1.387 (2)	C33—H33A	0.93
C15—H15A	0.93	C34—H34A	0.93
C16—H16A	0.93		
			
C2—N1—N5	118.83 (12)	H17B—C17—H17C	109.5
C2—N1—C1	108.47 (13)	C14—C18—C19	114.99 (13)
N5—N1—C1	132.69 (12)	C14—C18—H18A	108.5
C2—N2—N3	104.23 (12)	C19—C18—H18A	108.5
C1—N3—N2	113.84 (12)	C14—C18—H18B	108.5
C1—N3—C22	125.69 (13)	C19—C18—H18B	108.5
N2—N3—C22	120.35 (12)	H18A—C18—H18B	107.5
C23—N4—C29	119.80 (12)	C20—C19—C21	110.56 (13)
C23—N4—C22	121.01 (13)	C20—C19—C18	110.48 (12)
C29—N4—C22	119.14 (13)	C21—C19—C18	111.82 (13)
C3—N5—N1	118.00 (12)	C20—C19—H19A	107.9
N3—C1—N1	102.21 (12)	C21—C19—H19A	107.9
N3—C1—S1	127.43 (12)	C18—C19—H19A	107.9
N1—C1—S1	130.26 (12)	C19—C20—H20A	109.5
N2—C2—N1	111.21 (13)	C19—C20—H20B	109.5
N2—C2—C10	125.62 (13)	H20A—C20—H20B	109.5
N1—C2—C10	123.10 (13)	C19—C20—H20C	109.5
N5—C3—C4	119.71 (13)	H20A—C20—H20C	109.5
N5—C3—H3A	120.1	H20B—C20—H20C	109.5
C4—C3—H3A	120.1	C19—C21—H21A	109.5
C5—C4—C9	118.73 (14)	C19—C21—H21B	109.5
C5—C4—C3	118.76 (13)	H21A—C21—H21B	109.5
C9—C4—C3	122.39 (14)	C19—C21—H21C	109.5
C6—C5—C4	121.49 (14)	H21A—C21—H21C	109.5
C6—C5—H5A	119.3	H21B—C21—H21C	109.5
C4—C5—H5A	119.3	N4—C22—N3	112.04 (13)
C5—C6—C7	118.48 (14)	N4—C22—H22A	109.2
C5—C6—H6A	120.8	N3—C22—H22A	109.2
C7—C6—H6A	120.8	N4—C22—H22B	109.2
C6—C7—C8	121.48 (15)	N3—C22—H22B	109.2
C6—C7—Br1	119.31 (12)	H22A—C22—H22B	107.9
C8—C7—Br1	119.20 (12)	C28—C23—C24	118.75 (15)
C9—C8—C7	119.73 (14)	C28—C23—N4	119.42 (14)
C9—C8—H8A	120.1	C24—C23—N4	121.83 (14)
C7—C8—H8A	120.1	C25—C24—C23	119.77 (15)
C8—C9—C4	120.05 (14)	C25—C24—H24A	120.1
C8—C9—H9A	120.0	C23—C24—H24A	120.1
C4—C9—H9A	120.0	C26—C25—C24	120.90 (16)
C2—C10—C11	111.34 (12)	C26—C25—H25A	119.5
C2—C10—C17	110.33 (13)	C24—C25—H25A	119.5
C11—C10—C17	110.39 (12)	C25—C26—C27	118.88 (16)
C2—C10—H10A	108.2	C25—C26—H26A	120.6
C11—C10—H10A	108.2	C27—C26—H26A	120.6
C17—C10—H10A	108.2	C28—C27—C26	121.13 (16)
C12—C11—C16	118.23 (14)	C28—C27—H27A	119.4
C12—C11—C10	120.12 (13)	C26—C27—H27A	119.4
C16—C11—C10	121.44 (14)	C27—C28—C23	120.54 (15)
C13—C12—C11	120.94 (14)	C27—C28—H28A	119.7
C13—C12—H12A	119.5	C23—C28—H28A	119.7
C11—C12—H12A	119.5	C30—C29—C34	119.83 (14)
C12—C13—C14	121.13 (15)	C30—C29—N4	121.92 (14)
C12—C13—H13A	119.4	C34—C29—N4	118.25 (13)
C14—C13—H13A	119.4	C29—C30—C31	119.85 (14)
C15—C14—C13	117.60 (15)	C29—C30—H30A	120.1
C15—C14—C18	121.65 (14)	C31—C30—H30A	120.1
C13—C14—C18	120.75 (14)	C32—C31—C30	120.24 (15)
C16—C15—C14	121.29 (14)	C32—C31—H31A	119.9
C16—C15—H15A	119.4	C30—C31—H31A	119.9
C14—C15—H15A	119.4	C31—C32—C33	119.87 (15)
C15—C16—C11	120.77 (15)	C31—C32—H32A	120.1
C15—C16—H16A	119.6	C33—C32—H32A	120.1
C11—C16—H16A	119.6	C34—C33—C32	120.24 (15)
C10—C17—H17A	109.5	C34—C33—H33A	119.9
C10—C17—H17B	109.5	C32—C33—H33A	119.9
H17A—C17—H17B	109.5	C33—C34—C29	119.96 (14)
C10—C17—H17C	109.5	C33—C34—H34A	120.0
H17A—C17—H17C	109.5	C29—C34—H34A	120.0
			
C2—N2—N3—C1	−0.72 (16)	C11—C12—C13—C14	−0.1 (2)
C2—N2—N3—C22	175.64 (13)	C12—C13—C14—C15	1.3 (2)
C2—N1—N5—C3	159.63 (14)	C12—C13—C14—C18	−178.97 (14)
C1—N1—N5—C3	−21.9 (2)	C13—C14—C15—C16	−1.0 (2)
N2—N3—C1—N1	1.58 (16)	C18—C14—C15—C16	179.34 (14)
C22—N3—C1—N1	−174.54 (13)	C14—C15—C16—C11	−0.6 (2)
N2—N3—C1—S1	−175.19 (11)	C12—C11—C16—C15	1.8 (2)
C22—N3—C1—S1	8.7 (2)	C10—C11—C16—C15	−173.02 (14)
C2—N1—C1—N3	−1.81 (15)	C15—C14—C18—C19	91.60 (18)
N5—N1—C1—N3	179.61 (14)	C13—C14—C18—C19	−88.07 (18)
C2—N1—C1—S1	174.83 (12)	C14—C18—C19—C20	171.34 (13)
N5—N1—C1—S1	−3.7 (3)	C14—C18—C19—C21	−65.06 (17)
N3—N2—C2—N1	−0.53 (15)	C23—N4—C22—N3	−58.20 (18)
N3—N2—C2—C10	−177.61 (13)	C29—N4—C22—N3	119.16 (15)
N5—N1—C2—N2	−179.65 (12)	C1—N3—C22—N4	134.71 (15)
C1—N1—C2—N2	1.54 (17)	N2—N3—C22—N4	−41.18 (18)
N5—N1—C2—C10	−2.5 (2)	C29—N4—C23—C28	−28.3 (2)
C1—N1—C2—C10	178.71 (13)	C22—N4—C23—C28	149.07 (15)
N1—N5—C3—C4	−175.35 (12)	C29—N4—C23—C24	152.09 (15)
N5—C3—C4—C5	173.71 (14)	C22—N4—C23—C24	−30.6 (2)
N5—C3—C4—C9	−2.3 (2)	C28—C23—C24—C25	2.1 (2)
C9—C4—C5—C6	2.0 (2)	N4—C23—C24—C25	−178.29 (14)
C3—C4—C5—C6	−174.15 (14)	C23—C24—C25—C26	−1.6 (2)
C4—C5—C6—C7	−1.0 (2)	C24—C25—C26—C27	0.2 (2)
C5—C6—C7—C8	−0.5 (2)	C25—C26—C27—C28	0.7 (3)
C5—C6—C7—Br1	178.70 (11)	C26—C27—C28—C23	−0.2 (3)
C6—C7—C8—C9	1.0 (2)	C24—C23—C28—C27	−1.2 (2)
Br1—C7—C8—C9	−178.16 (11)	N4—C23—C28—C27	179.18 (15)
C7—C8—C9—C4	−0.1 (2)	C23—N4—C29—C30	121.59 (16)
C5—C4—C9—C8	−1.4 (2)	C22—N4—C29—C30	−55.8 (2)
C3—C4—C9—C8	174.57 (14)	C23—N4—C29—C34	−58.07 (19)
N2—C2—C10—C11	103.74 (17)	C22—N4—C29—C34	124.55 (15)
N1—C2—C10—C11	−73.02 (18)	C34—C29—C30—C31	−0.7 (2)
N2—C2—C10—C17	−19.2 (2)	N4—C29—C30—C31	179.60 (14)
N1—C2—C10—C17	164.05 (13)	C29—C30—C31—C32	0.7 (2)
C2—C10—C11—C12	135.56 (14)	C30—C31—C32—C33	0.1 (2)
C17—C10—C11—C12	−101.54 (16)	C31—C32—C33—C34	−1.0 (2)
C2—C10—C11—C16	−49.71 (19)	C32—C33—C34—C29	1.0 (2)
C17—C10—C11—C16	73.20 (17)	C30—C29—C34—C33	−0.1 (2)
C16—C11—C12—C13	−1.5 (2)	N4—C29—C34—C33	179.55 (14)
C10—C11—C12—C13	173.45 (14)		

### Hydrogen-bond geometry (Å, °)

**Table:**

*D*—H···*A*	*D*—H	H···*A*	*D*···*A*	*D*—H···*A*
C3—H3A···S1	0.93	2.52	3.217 (2)	132
C22—H22B···S1	0.97	2.80	3.232 (2)	108
C6—H6A···Cg1^i^	0.93	2.81	3.717 (2)	165
C21—H21C···Cg2^ii^	0.96	2.89	3.829 (2)	168

Symmetry codes: (i) −*x*+1, −*y*, −*z*; (ii) −*x*+1, *y*+1/2, −*z*+1/2.
